# Inguinal Colotomy

**Published:** 1890-03

**Authors:** 


### INGUINAL COLOTOMY.

Mr. Harrison Cripps, in the discussion on the treatment of cancer on the rectum, at the recent meeting of the British Medical Association (*British Medical Journal*, October 12, 1889), reiterated his well-known views as to the advantages of the intra-peritoneal or inguinal method of opening the colon, over the lumbar or extra-peritoneal procedure. He summarizes them as follows:

1. The room in front in which the operator has to work is practically unlimited, and he is not hampered by working in the narrow space between the last rib and the crest of the ilium, as in lumbar colotomy. The bowel can be exposed by a clean incision; the extensive bruising and laceration of the sub-peritoneal fat, often quite unavoidable when searching for the colon from behind, are thus prevented.

2. It is far easier absolutely to identify the bowel from in front, and it is scarcely possible to make the mistake of opening the small intestine, duodenum, or stomach, which not infrequently happens in the lumbar method.

3. In a fat or muscular patient, the bowel can be fixed to the skin with much less tension, partly owing to the mobility of the sigmoid flexure, and partly to the ease with which the skin can be depressed.

4. If the bowel takes an abnormal course, it will scarcely affect the operation, while such a deviation in the lumbar region leads to complete failure.

He makes his incision rather higher than most surgeons, its centre being a little above an imaginary line drawn from the anterior superior spine to the umbilicus. The incision crosses this line at right angles two inches from the anterior spine. As soon as the colotomy wound has consolidated, the rectum may be washed out once daily with weak Condy's fluid, followed by a boracic lotion, ten grains to the ounce.—*American Journal Medical Sciences.*

